# Comparative Genomics and Phenotypic Characterization of *Gluconacetobacter entanii*, a Highly Acetic Acid-Tolerant Bacterium from Vinegars

**DOI:** 10.3390/foods12010214

**Published:** 2023-01-03

**Authors:** Karin Jelenko, Eva Cepec, Francisco X. Nascimento, Janja Trček

**Affiliations:** 1 Department of Biology, Faculty of Natural Sciences and Mathematics, University of Maribor, 2000 Maribor, Slovenia; 2 iBET, Instituto de Biologia Experimental e Technológica, 2781-901 Oeiras, Portugal; 3 Faculty of Chemistry and Chemical Engineering, University of Maribor, 2000 Maribor, Slovenia

**Keywords:** acetic acid bacteria, *Gluconacetobacter*, *Novacetimonas*, *Gluconacetobacter entanii*, *Komagataeibacter*, vinegar, acetic acid tolerant bacteria

## Abstract

The bacterial species *Gluconacetobacter entanii* belongs to a group of acetic acid bacteria. In 2000, it was described as a primary species of submerged spirit vinegar-producing bioreactors with a strict requirement of acetic acid, ethanol, and glucose for growth. Over the years, the type-strain of *G. entanii* deposited in international culture collections has lost the ability for revitalization and is thus not available any more in a culturable form. Here, we have systematically characterized phenotypic features and genomes of recently isolated *G. entanii* strains and compared them with characteristics of the type-strain available from published data. Using the functional annotation, genes *gmhB* and *psp* were identified as unique for *G. entanii* genomes among species in the clade *Novacetimonas*. The genome stability of *G. entanii* was assessed after 28 and 43 months of preculturing the strain *Gluconacetobacter entanii* AV429 twice a week. The strain *G. entanii* AV429 did not accumulate giant insertions or deletions but a few gene mutations. To unify further research into acetic acid bacteria systematics and taxonomy, we propose *G. entanii* AV429 as the neotype strain.

## 1. Introduction

*Gluconacetobacter entanii* was described as a novel bacterial species in a group of acetic acid bacteria in 2000 [1]. It was isolated from submerged high acetic acid-containing spirit vinegar bioprocesses in the Southern part of Germany. The phenotypic analysis of four strains revealed their extraordinary characteristics: the inability to grow in the absence of acetic acid, ethanol, and glucose, the requirement of total concentrations of acetic acid plus ethanol higher than 6% for growth, and the inability to over-oxidize acetic acid to CO_2_ and H_2_O. The strains evolved extreme adaptation to high concentrations of acetic acid, as high as 11 vol%, being, to our knowledge, the microorganism with the highest tolerance to acetic acid ever described. According to the taxonomic requirements for novel species description, one of these strains has been described as a type-strain and deposited into international microbial culture collections [1].

Although much attention has been given to developing an improved long-term preservation protocol for *G. entanii* strains [2], the type-strain available from the German collection of microorganisms (DSMZ) lost its ability to be recovered on growth media from the preserved form. Today, the type-strain of *G. entanii* is not available in a culturable form from any of the publicly available bacterial culture collections [3,4]. This is also why this species kept the naming *Gluconacetobacter entanii* although the clade to which *G. entanii* taxonomically belongs has been in 2013 reclassified into *Komagataeibacter* genus [5,6] and in 2022 into the novel genus *Novacetimonas* [4]. Besides *G. entanii*, the clade includes *Novacetimonas hansenii*, *Novacetimonas maltaceti*, *Novacetimonas pomaceti* and *Novacetimonas cocois*. However, although the type-strain is lost, its genomic DNA was preserved for genome sequencing. The genome sequence of the *G. entanii* type strain is thus publicly available, which enables comparative analysis with genome sequences of the other strains [7].

The *G. entanii* species was also mentioned in other scientific papers after its taxonomic description. In 2007, a strain isolated from vinegar in Mexico was, after 16S rRNA analysis, identified as *G. entanii* [8]. After growth on different carbon sources, metabolic pathways were reconstructed for this strain, and later in 2019, it was successfully used for cellulose production on a nutshell of pecan [9]. A publication from 2014 mentions another *G. entanii* strain, ACCC10215, isolated from rowans for cellulose production [10]. Since the *G. entanii* species presents biotechnological potential, we aim to elucidate its features further by comparative genome analysis and growth characteristics. Due to the loss of the *G. entanii* type strain, we explore the possibility of proposing one of the here-described strains as a neotype strain of *G. entanii* that would replace the type strain.

## 2. Materials and Methods

### 2.1. Morphological, Biochemical and Physiological Characterization of G. entanii

Bacterial strains used in this study are listed in Table 1. The strains have been revitalized from −80 °C on RAE medium (glucose 40 g/L, peptone 10 g/L, yeast extract 10 g/L, citric acid 1.37 g/L, Na_2_HPO_4_ · 2 H_2_O 3.38 g/L, agar 10 g/L) containing glacial acetic acid (1 vol%) and absolute ethanol (1 vol%) with incubation at 30 °C and 92–96% relative air humidity for 3 days [2,7].

The phenotypic characterization of strains *G. entanii* AV429, *G. entanii* SI2084 and *G. entanii* FXV2 was principally performed as described previously [7]. Briefly, growth on various carbon sources was tested in liquid medium containing 1% of the selected carbon source, 0.5% of yeast extract, and pH adjusted to 6.8. The growth was positive if A_600_ reached at least 0.3 in 7 days. An A_600_ between 0.1 and 0.3 was designated as weak growth. Growth in the presence of 30% glucose (5 g/L yeast extract, 300 g/L glucose and 15 g/L agar) was tested during two-weeks incubation at 30 °C. Growth in the presence of D-glucose, D-mannitol and ethanol as a sole carbon source in the presence of ammonium sulphate as the sole nitrogen source was tested on Hoyer–Frateur and Asai culture media during ten days of incubation at 30 °C. Growth in the presence of different ethanol and acetic acid concentrations was tested in liquid RAE medium containing 1 vol% ethanol and 1.0–7.0 vol% of acetic acid or RAE medium containing 3 vol% ethanol and 1.0–7.0 vol% of acetic acid. Additionally, tests specifically described for *G. entanii* 4560^T^ by Schüller et al. [1] were performed as described above except that AE medium (2 g/L yeast extract, 3 g/L peptone and 5 g/L of the selected carbon source) with addition of 4 vol% acetic acid and 3 vol% ethanol was used as a growth medium. Gluconic acids were identified with a modified method of Gosselé et al. [11] as described by Marič et al. [12]. The presence of cellulose was checked in 5% NaOH, as described by Navarro et al. [13].

Comparison of lag phase and specific growth rates among strains AV429, SI2084 and FXV2 was estimated from growth curves in an AE medium containing 4 vol% of acetic acid and 3 vol% of ethanol generally as described previously [14]. More specifically, a 250-mL baffled flask containing 50 mL AE broth with acetic acid and ethanol was inoculated with 500 μL of bacterial culture with a density of 0.4 at A_600_ and incubated at 30 °C and 180 rpm. The bacterial growth was monitored by the optical density at A_600_. The exponential growth phase was identified in log(A_600_) vs. time plot. The specific growth rates (μ) were calculated by linear regression of ln(A_600_) vs. time, with growth rate as the regression coefficient. All strains grew in this medium planktonically.

For genome stability experiments, strain *G. entanii* AV429 was precultured on an RAE medium containing 1 vol% of ethanol and 1 vol% of acetic acid twice a week for 43 months. For further analysis, DNA extracted from the biomass harvested after 28 months (strain designation *G. entanii* AV429-2020) and 43 months (*G. entanii* AV429-2022) was used.

**Table 1:** List of strains used in the study.

Strain Designation	Source and Country of Isolation or Other Features	Reference
*Gluconacetobacter entanii* AV429	Apple cider vinegar (Slovenia)	[15]
*Gluconacetobacter entanii* AV429-2020	Strain AV429 precultured for 28 months	This study
*Gluconacetobacter entanii* AV429-2022	Strain AV429 precultured for 43 months	This study
*Gluconacetobacter entanii* FXV2	Fermented grape must (Portugal)	[4]
*Gluconacetobacter entanii* SI2084	Apple cider vinegar (Slovenia)	[16]
*Gluconacetobacter entanii* KS542	Apple cider vinegar (Slovenia)	In-house strain
*Gluconacetobacter entanii* KS544	Apple cider vinegar (Slovenia)	In-house strain
*Gluconacetobacter entanii* KS545	Apple cider vinegar (Slovenia)	In-house strain

### 2.2. Genome Sequences, Assembly and Annotation

Whole genome sequencing of strains *G. entanii* KS542, *G. entanii* KS544, *G. entanii* KS545, *G. entanii* AV429-2020 and *G. entanii* AV429-2022 was performed at the Department for Microbiology, Faculty of Medicine, University of Maribor. The DNA of the strains was isolated from the exponential growth phase using the GeneJET Genomic DNA Purification Kit (Thermo Scientific, Waltham, MA, USA) and subjected to genome sequence analysis. The paired-end libraries were prepared with the Nextera XT DNA Library Preparation Kit (Illumina, San Diego, CA, USA) following the manufacturer’s protocol. The sequencing was performed on Illumina NextSeq2000 (Illumina, San Diego, CA, USA). Fastq reads were quality-checked and trimmed with Trimmomatic [17], followed by the genome assembly using SPAdes version number 3.13.1 (https://cab.spbu.ru/software/spades/) [18] with default parameters and the option “careful”; the option tries to reduce the number of mismatches and short indels. The genome sequences were deposited into the GenBank database under the accession numbers presented in Table 2. The accession numbers for strains *G. entanii* KS542, *G. entanii* KS544, *G. entanii* KS545, *G. entanii* AV429-2020, and *G. entanii* AV429-2022 are JANGSU000000000, JANGST000000000, JANGSS000000000, JANGSR000000000, JANGSQ000000000, respectively. Assembly was performed at the NCBI using the Prokaryotic Genome Annotation Pipeline (PGAP).

### 2.3. Phylogenomic Studies

The overall genome similarities among *G. entanii* strains were measured by orthologous average nucleotide identity algorithm (OrthoANI) using EZBioCloud [19]. For each sequenced genome, contigs longer than 500 bp were extracted and used for OrthoANI calculation. Genome distances between *G. entanii* strains were calculated by the Genome-to-Genome Distance Calculator 2.1. The method reliably mimics conventional DNA-DNA hybridization [20].

A phylogenetic tree was constructed using concatenated core genes. The procedure started with local annotation of genome sequences by Prokka [21], followed by alignment at the minimum BlastP [22] identity of 90% by Roary [23]. From the concatenated core genes the phylogeny was inferred with the maximum likelihood algorithm included in PhyML [24] using the GTR nucleotide substitution model.

### 2.4. Comparative Genomic Analysis

A primary search for insertion sequences (IS) in the genomes of different *G. entanii* strains was performed by the program ISfinder [25]. The following criteria were further used to identify and confirm the presence and the number of homologous IS-elements by using program Geneious Prime [26]: E value below 10^−50^, query coverage over 40%, and pairwise nucleotide identity over 60%. In the strain *G. entanii* AV429, each of the identified IS-elements was further followed through sequence analysis of the precultured strain.

The presence of putative plasmids in *G. entanii* genome sequences was assessed from the presence of Rep proteins in their genome sequences. First, a database of Rep proteins originating from *Acetobacter*, *Komagataeibacter*, and *Novacetimonas* plasmid sequences available in NCBI was constructed. To identify identical proteins, the Rep proteins were aligned by ClustalX. Proteins differentiating in at least one amino acid were used as queries for searching homologues in the *G. entanii* genomes applying the same criteria as for the IS elements, except that the minimal requirement for amino acid identity was set at 40%.

Distribution of prophages in *G. entanii* genomes was evaluated by the Phaster webbased tool [27]. The identified prophages were categorized as intact, questionable, and incomplete depending on the representation of phage proteins in the bacterial genome. The intact prophages were further annotated using RAST server [28].

To identify specific genes in *G. entanii* strains, the functional annotation of the genomes was conducted by GhostKOALA [29]. The genomes of the strains were then categorized based on the presence/absence of KOs (abbreviation for KEGG Orthology), which were searched using a python script [4]. In this way, the KOs which exist in all *G. entanii* strains and are absent from all *Novacetimonas* strains of which genomes are available at NCBI were identified. In a second approach, marker genes for *G. entanii* were selected based on KO existence in 75% of the *G. entanii* strains and the absence of KO in more than 25% of *Novacetimonas* strains.

Principal Component Analysis (PCA) was applied to the functional annotation data. More precisely, BlastKOALA was used to annotate the CDSs of each strain with KO identifiers. Each genome and its set of annotated KOs were then used in a PCA analysis using the SIMCA 17. 0. 1 (Goettingen, Germany). This way, the strains based on the KO presence/absence and KO copy number were grouped. Strains with similar functional annotations tended to group closer and vice versa. The number of principal components used for the modeling was optimized by leave-one-observation-out cross-validation.

## 3. Results and Discussion

### 3.1. Phylogenomic Studies

The genomes of *G. entanii* strains LTH 4560^T^, AV429, FXV2, SI2084, KS542, KS544, and KS545 were subjected to phylogenomic analysis. The orthoANI values among these strains ranged from 98.31% to 100% (Table S1). These results confirm that all these strains belong to the species *Gluconacetobacter entanii* since all values are above the 95–96% cut-off value for species demarcation [30]. Besides, the four strains SI2084, KS542, KS544, and KS545 exhibit very high similarities, from 99.8% to 100%. These strains were isolated from the same bioreactor for apple cider vinegar production [16], suggesting their clonal relatedness. The strains LTH 4560^T^, AV429, and FXV2 have different geographical origins and isolation sources (Table 1). The OrthoANI values among these strains and one of the strain mentioned above, SI2084, ranged from 98.33% to 98.60%, confirming their genetic differences.

The in silico DNA-DNA hybridization among genomes of *G. entanii* strains (Table S2) coincides with the findings of the orthoANI analyses. All values are above 70%, being a threshold for strain allocation into the same species [20]. Besides, high genome to genome similarities among the strains SI2084, KS542, KS544, and KS545 are confirming that the strains are of the same clonal group.

**Figure 1:**
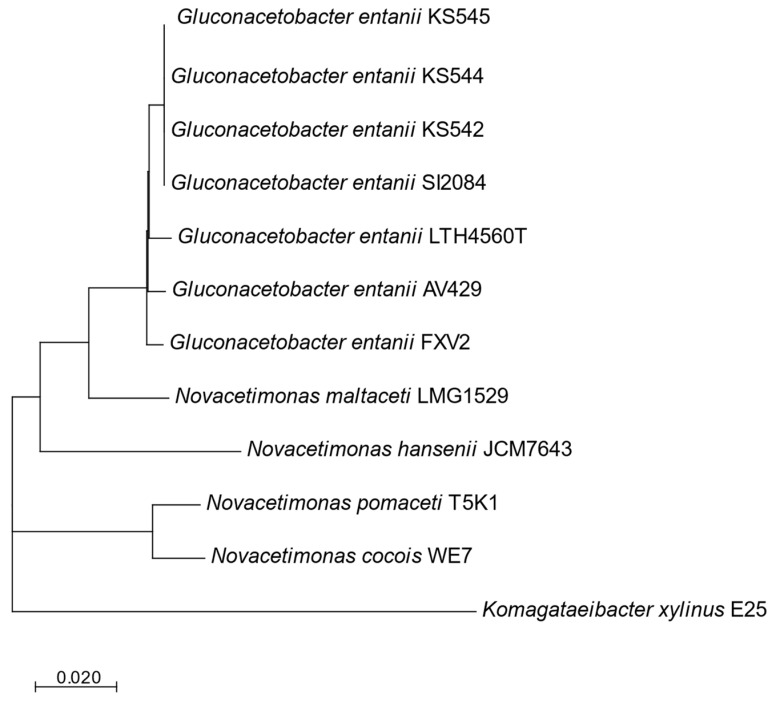
Phylogenetic reconstruction based on core genes (238 at minimum BlastP identity of 90%) of the *Gluconacetobacter entanii* strains and the type strains of *Novacetimonas* species. The tree was constructed using the maximum-likelihood method. The scale bar represents the number of substitutions per site.

A phylogenetic tree based on 238 core genes groups all *G. entanii* strains into a single subclade of the clade encompassing the genus *Novacetimomas*. Besides *G. entanii*, the clade includes species *N. hansenii*, *N. cocois*, *N. pomaceti*, and *N. maltaceti* (Figure 1).

### 3.2. Morphological, Biochemical and Physiological Characterization of G. entanii

All strains were Gram-staining negative rods, in length 1.79–3.88 μm and width 0.7–1.11 μm as determined by microscopy with 1000 magnification. They were all catalase-positive and oxidase-negative. Further phenotypic analysis was performed with the strains AV429, SI2084, and FXV2, which were, as described above, identified as distinctively genomically different.

To determine if the strains are similar to the type strain *G. entanii* LTH 4560^T^, the phenotypic characterization was performed in AE medium containing 4% acetic acid and 3% ethanol. As presented in Table 3, only two phenotypic characteristics, typical for the type strain, matched all strains of *G. entanii* studied here. Additionally, the phenotypic characteristics usually analyzed for other acetic acid bacteria [7] have been analyzed for the G. entanii strains. In this case, the characteristics also differ among strains (Table 4).

**Figure 2:**
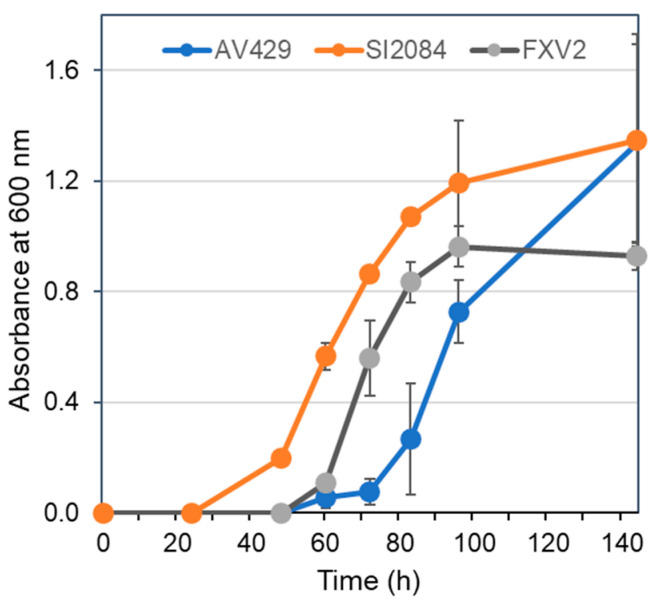
Growth curves of strains *G. entanii* AV429, *G. entanii* SI2084 and *G. entanii* FXV2 in medium AE containing 4 vol% of acetic acid and 3 vol% of ethanol.

A comparison of the growth curves (Figure 2) among *G. entanii* strains (AV429, SI2084, FXV2) in liquid medium AE containing 4% acetic acid and 3% ethanol revealed differences among the strains in the length of the lag phase but similar specific growth rates. Among the three *G. entanii* strains, the strain SI2084 has the shortest adaptation time to 4% of acetic acid.

**Figure 3:**
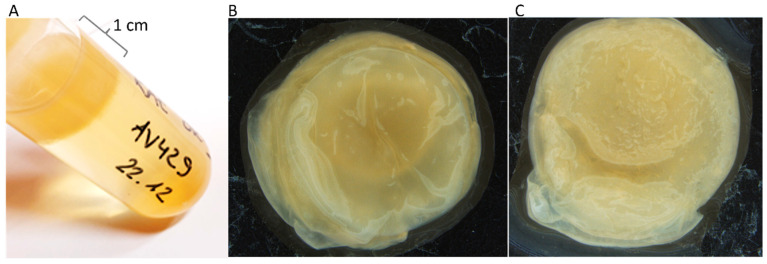
*Gluconacetobacter entanii* AV429 forms a thick pellicle at the top of the RAE medium containing 1 vol% ethanol and 2.5 vol% acetic acid (**A**). The pellicle picture was taken from the top (**B**) and from the bottom (**C**). Biofilm images were taken by microscopy with 6.3 magnification.

The strains AV429 and FXV2 form a very thick cellulose pellicle (~1 cm) in an RAE medium containing 1% acetic acid and 1% ethanol after 14 days of cultivation (data not shown). A similar pellicle was detected with the strain AV429 also in medium containing 2.5% acetic acid and 1% ethanol after 12 days. The pellicle was composed of layers combined with filamentous structures. It looks smooth from the top and structured from the bottom (Figure 3). The formation of such a thick cellulose pellicle in a few days is an interesting bacterial characteristic for biomaterial production with potential applications in medicine, cosmetics, the food industry, and electronics.

### 3.3. Basic Genome Characteristics

The basic characteristics of the four *G. entanii* genomes are shown in Table 2. The features are very similar among the strains. In comparison to other acetic acid bacteria [7,31], the genome size of *G. entanii* strains (approx. 3.65 Mbp) is somewhere in the middle, as the smallest genome *K. kakiaceti* has size of 3.13 Mbp and the biggest *K. europaeus* of size 4.22 Mbp. The G + C content is 62.5% or 62.6%, which is in the range of other acetic acid bacteria [7]. However, this number is rather high compared to other bacteria, given the relatively small genome size. For example, *E. coli*, which also represents a bacterium that adapts to a free-living environment, has a G + C content of only approximately 50%. However, its genome size is bigger, 4.6–5.5 Mbp [32]. This suggests that in the genomes of *G. entanii* and other acetic acid bacteria, a higher number of horizontally transmitted genes, such as transposases, drug-resistance genes, and others, such as prophages and genes encoding transporters, have accumulated through their evolution [33]. The increased G + C content also follows previous findings that aerobic prokaryotes generally possess a higher G + C% compared to anaerobic prokaryotes [34]. The proportion of pseudogenes varies between 2.9% and 4.7%, which is similar to *E. coli* and other non-pathogenic, non-intracellular, and non-symbiotic bacteria [35].

### 3.4. Mobilome Analysis

Plasmids represent one of the mobile elements in bacteria and the primary vector of horizontal gene transfer. They are essential for conferring diversity and evolution of bacteria. In the NCBI database there is presently only one strain of the genus Novacetimons (Novacetimonas hansenii C110) available with plasmid sequences. The plasmid profile of *G. entanii* LTH4560^T^ presented in the original paper also indicates a presence of more plasmids. To gain information into the plasmid repertoire of *G. entanii* strains, we built a database of Rep proteins from plasmids available in the NCBI database for species of *Acetobacter*, *Gluconacetobacter*, and *Novacetimonas*. Using rigorous criteria, we searched the homologues in the genomes of *G. entanii*. We identified the highest number of Rep proteins differentiating in at least one amino acid in *G. entanii* LTH4560^T^, followed by *G. entanii* SI2084, *G. entanii* AV429, and *G. entanii* FXV2 (Table S3). Although this data cannot directly correlate with the number of plasmids, since the same plasmid may possess more Rep proteins and the plasmid might be part of the chromosome, the results confirm genomic diversity among the strains. In addition, the results indicate a relatively high number of putative plasmids in these strains. Further genome sequencing is necessary to obtain complete closed genomes, thus enabling precise analysis on the number of conjugative, mobilizable, and non-mobilizable plasmids in G. entanii strains.

Insertion sequences (IS) are another widespread mobile element in bacteria. Their classification is based on the transposases that catalyze their movement through the genome. The genomes can have a significant number of truncated and partial IS-elements without transposases. Here we have searched only the entire IS-elements. We identified the highest number of the whole IS-elements in type strain *G. entanii* LTH4560^T^, followed by *G. entanii* SI2084, *G. entanii* AV429, and *G. entanii* FXV2 (Table 5). None of these IS elements were present in all *G. entanii* strains. In *G. entanii* LTH4560^T^, two IS-elements, IS*Gxy*1 and IS*1031*A, were identified in four copies. The IS*1031*A has been previously associated with the inactivation of cellulose production [36].

Prophages are another type of mobile bacterial element. They promote gene transfer in and among bacterial populations [37,38]. A recent study revealed phage-like particles in 15 of 177 acetic acid bacterial strains. They all show morphology similar to *Myoviridae*-type phage [39]. Here we have identified one or two types of complete prophages and up to five incomplete prophages in *G. entanii* strains (Table 6). Further investigation of these genetic elements revealed that the sequences contain the putative gene for phage terminase large subunit; one is similar to a homologue of *Komagataeibacter* phage phiKM1 (BCZ75968.1) and the other to a homologue of phage of *Myoviridae* sp. (DAQ38419.1) identified in the human metagenome (Table 7). One type of prophage identified here shows very high overall nt-identity to *Komagataeibacter* phage phiKM1, the phage of the family *Myoviridiae* (Table 7).

### 3.5. A Comparative Genomic Functional Analysis

The functional annotation of nine *G. entanii* strains and 15 strains of *Novacetimonas* species, including all presently recognized type strains, resulted in the prediction of a total number of 1741 KOs, of which three were unique for *G. entanii* (Table 8), and six were present in 75% of *G. entanii* and less than 25% of *Novacetimonas* strains (Table 9). Of the three unique genes, the *gmhB* is coding for a putative D-glycero-D-manno-heptose 1,7-bisphosphate phosphatase. The gene and its product were characterized in some bacteria, from which we know it to be involved in the biosynthesis of the ADP-L-glycerol-ß-D-manno-heptose precursor of the inner core lipopolysaccharide (LPS) [40,41,42,43]. According to this data, this component is a unique component of the *G. entanii* LPS in the clade encompassing *Novacetimonas* species. This component may be involved in high acetic acid resistance. The second unique gene *psp* of *G. entanii* codes for a putative phosphoserine phosphatase with the highest query cover (83%) and amino-acid-identity (29.8%) to the phosphoserine phosphatase (PSP) of *Hydrogenobacter thermophilus,* representing a novel type of PSP [44]. The enzyme is supposed to catalyze the dephosphorylation of phosphoserine to serine and inorganic phosphate as part of the serine pathway in microorganisms. However, this particular type of PSP had been annotated as cofactor-dependent, possesses neither mutase activity nor the residues important for the activity, and has been since 2012 consequently defined as a novel-type PSP [44]. The third gene identified in this clade and being specific for *G. entanii* has no significant identity to any characterized protein.

Interestingly, the *G. entanii* genomes have, on average, an additional four copies of *parA*, three copies of *parB*, and three copies of *hupB*, when compared to *Novacetimonas* spp. (Tables S4 and S5). The primary role of ParA and ParB in bacteria is the segregation of newly replicated DNA [45,46]. For the HupB, it has been suggested to be involved in coordinating replication with DNA segregation [47]. All these findings support our above mentioned observation that *G. entanii* strains may possess more plasmids. Interestingly, significantly overrepresented in *G. entanii* strains with three additional copies on average is also *gabD*, putatively coding for succinate-semialdehyde dehydrogenase, which may be involved in glutaric acid production from lysin [48].

**Figure 4:**
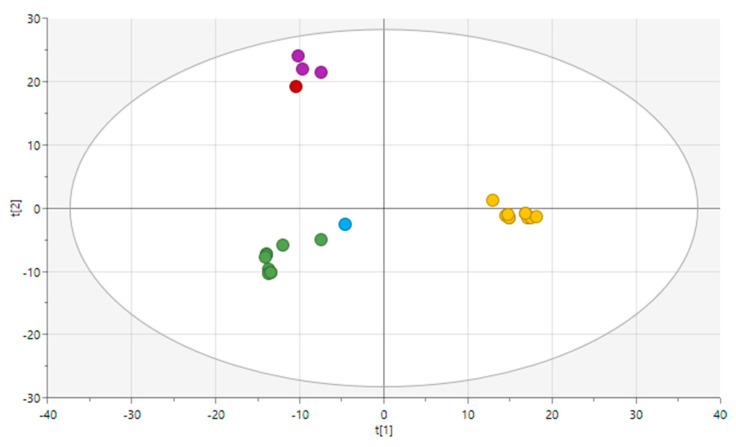
PCA analysis based on the functional annotation (KO copy number and prevalence) of strains from the clade *Novacetimonas*. A total of 1741 KOs were used. Legend: green, *G. entanii*; blue, *N. maltaceti*; purple, *N. pomaceti*; red, *N. cocois*; yellow, *N. hansenii*.

The PCA model with two principal components explains 54% of the variance present in the functional annotation data. Figure 4 shows the score plot for the two components of the PCA model, showing three clusters of observations, which corroborates the results obtained in the phylogenomic and core gene analysis, grouping together closely related strains. The obtained results indicate that the functional genomic properties of the strains are intimately related to their phylogeny.

### 3.6. Genome Stability of G. entanii AV429

Several papers on acetic acid bacteria have reported on the genetic instability of acetic acid bacteria, mainly due to the presence of transposons and plasmids [49,50]. To check the genome stability of *G. entanii*, the strain AV429 was systematically precultured for several months on an agar medium containing 1% ethanol and 1% acetic acid. After 43 months of preculturing, no giant insertions, or deletions happened (Figure S1), which is probably a result of keeping the strain under non-harsh conditions, but rather in an environment with high glucose content and moderate concentrations of ethanol and acetic acid; this resembles conditions during vinegar production, from where most of the presently identified *G. entanii* strains originate. A very few gene mutations have been identified, such as in genes coding efflux pump membrane protein, dehydrogenases, transposases, and the others, whose potential effects are yet to be identified.

## 4. Conclusions

Here, we have explored the characteristics of novel *G. entanii* strains and compared them with those described for the type-strain. The three novel *G. entanii* strains of different clonal origins have highly similar genome characteristics. However, their phenotypic traits, which are usually analyzed for taxonomic purposes in the group of acetic acid bacteria, differ substantially. Recently, novel recommendations have been introduced into bacterial taxonomy, giving solid weight to genome analysis [51]. The *G. entanii* genomes presented here undoubtedly show high similarity to the genome sequence of the type-strain, which is unavailable in a culturable form from any of the publicly available culture collections. Two species-specific marker genes, *gmhB* and *psp*, were identified in all presently known *G. entanii* genomes. In the era of affordable genome sequencing, these two genes allow us quick differentiation of this species from other validly published species in the clade *Novacetimonas*.

Since we need a culturable strain to unify the taxonomic research into *G. entanii*, we propose the *Gluconacetobacter entanii* AV429 (ZIM B1055, LMG 31305, CCM 8957) as a neotype strain for the species *Gluconacetobacter entanii*. This strain was genetically stable during preculturing for at least 43 months. The recognition of our proposal will enable the renaming of *Gluconacetobacter entanii* to *Novacetimonas entanii*, thus resolving the peculiar position of *G. entanii* in the *Novacetimonas* clade.

## Figures and Tables

**Table 2:** Comparison of general genome features of *G. entanii* strains.

Species	Accession Number	Number of Bases (Mbp)	G + C%	Genes	Proteins	rRNA	tRNA	Pseudogenes (% of Total Genes)
*Gluconacetobacter entanii* LTH4560^T^	NKUF00000000	3.60	62.6	3444	3243	5	50	142 (4.1)
*Gluconacetobacter entanii* AV429	JABJWD000000000	3.75	62.6	3523	3361	3	46	109 (3.1)
*Gluconacetobacter entanii* SI2084	JAILXQ000000000	3.64	62.6	3560	3339	3	48	166 (4.7)
*Gluconacetobacter entanii* FXV2	WNJT00000000	3.61	62.5	3135	2990	5	48	88 (2.9)

**Table 3:** Utilization of different carbon sources in AE (4aa/3e), AE (3e) and AE (4aa) broth by *Gluconacetobacter entanii* strains.

Carbon Source	LTH 4560^T^	LTH 4637	AV429	SI2084	FXV2
AE (4a/3e) broth	+	+	+	+	+
AE (4aa/3e) broth without glucose	−	−	+	W	+
AE (4aa/3e) broth; glucose replaced with:					
Maltose	+	+	W	W	W
Sucrose	+	+	+	+	+
Sorbitol	W	+	+	+	+
Mannitol	−	W	+	+	+
Lactate	−	−	+	+	W
Gluconate	−	−	+	W	−
Fructose	+	+	W	+	−
Glycerol	−	−	+	W	+
AE (3e) broth	−	−	W	−	W
AE (3e) broth; acetic acid replaced with:					
Lactate	−	−	+	−	−
Gluconate	−	−	+	−	−
AE (4aa) broth	−	−	+	W	−
AE (4aa) broth with 1-propanol	+	+	−	−	−

Legend: 4aa, 4 vol% acetic acid; 3e, 3 vol% ethanol; +, growth; −, no growth; W, weak growth.

**Table 4:** Phenotypic characteristics of *Gluconacetobacter entanii* strains.

Phenotypic Characteristics	Strain			
	LTH 4560^T^	AV429	SI2084	FXV2
Formation from D-glucose				
2-Keto-D-gluconic acid	−	+	+	+
5-Keto-D-gluconic acid	−	+	+	+
Growth on carbon sources:				
D-Ribose	n.k.	W	W	W
Sorbitol	n.k.	+	+	+
D-Mannitol	n.k.	+	+	+
Glycerol	n.k.	+	W	+
1-Propanol	n.k.	W	W	W
Growth in the presence of 30% D-Glucose	−	+	−	+
Utilization of ammoniacal nitrogen in:				
Hoyer–Frateur medium with				
D-Glucose	n.k.	+	+	+
D-Mannitol	n.k.	−	W	−
Ethanol	n.k.	+	+	W
Asai medium with				
D-Glucose	n.k.	+	−	−
D-Mannitol	n.k.	+	−	−
Ethanol	n.k.	+	+	W
Growth without acetic acid	−	+	+	W
Growth on RAE medium in the presence of 1% ethanol and acetic acid at:				
4%	n.k.	+	+	+
5%	n.k.	W	W	+
6%	n.k.	−	W	+
7%	n.k.	−	W	−
Growth on RAE medium in the presence of 3% ethanol and acetic acid at:				
4%	n.k.	+	+	+
5%	n.k.	−	+	+
6%	n.k.	−	+	−
7%	n.k.	−	+	−

Legend: n.k., not known; W, weak growth; +, good growth; −, no growth.

**Table 5:** Distribution of IS-elements among *Gluconacetobacter entanii* strains.

IS-Element (Length)	*G. entanii* LTH4560	*G. entanii* AV429	*G. entanii* SI2084	*G. entanii* FXV2
IS*Gxy*1 (1313 bp)	4	2	-	1
IS*1452* (1411 bp)	2	1	1	-
IS*1031*A (930 bp)	4	-	-	-
IS*1031*C or D (930 bp)	3	-	-	-
IS*Gdi*13 (1452 bp)	2	-	1	-
Tn*5393* (5470 bp)	1	-	1	-
IS*1032* (916 bp)	-	1	-	1
IS*Gdi*8 (1356 bp)	-	-	1	-
IS*Ppa*1 (1376 bp)	-	-	1	-
IS*Gdi*11 (1200 bp)	-	-	-	1

**Table 6:** Number of prophages identified in *Gluconacetobacter entanii* strains.

Strain Designation	Intact	Incomplete	Questionable
*Gluconacetobacter entanii* AV429	0	2	2
*Gluconacetobacter entanii* AV429-28	1	2	0
*Gluconacetobacter entanii* AV429-43	1	5	0
*Gluconacetobacter entanii* FXV2	0	2	0
*Gluconacetobacter entanii* SI2084	2	0	0
*Gluconacetobacter entanii* KS542	2	0	0
*Gluconacetobacter entanii* KS544	1	0	1
*Gluconacetobacter entanii* KS545	1	0	1

**Table 7:** Prophage identification in *Gluconacetobacter entanii* genomes using web-based tool Phaster.

Bacterial Host Strain	Node Length (kbp)/Node no.	Total Proteins of the Node/Phage Specific Proteins	Two Most Common Phage Species (acc. no.)	NCBI BlastN Similarity Results (acc.no.): Query Cover (%)/nt-Identity (%)
*Gluconacetobacter entanii* AV429-28	27.3/(node 3)	32/13	*Escherichia coli* phage Stx2a_F451 (NC_049924)	*Komagataeibacter* phage phiKM1 (LC644974.1): 43/95.7
			*Escherichia coli* phage Stx2_1717 (NC_011357)	-
*Gluconacetobacter entanii* AV429-43	26.0/(node 4)	30/13	*Erwinia* phage ENT90 (NC_019932)	*Komagataeibacter* phage phiKM1 (LC644974.1): 45/95.7
			*Burkholderia* phage phi644-2 (NC_009235)	-
*Gluconacetobacter entanii* SI2084	22.9/(node 145)	29/21	*Shigella* phage SfII (NC_021857)	*Komagataeibacter* phage phiKM1 (LC644974.1): 94/93.7
			*Enterobacteria* phage SfI (NC_027339)	-
	39.9/(node 166)	57/32	*Vibrio* phage martha 12B12 (NC_021070)	*-*
			*Escherichia* phage D108 (NC_013594)	-
*Gluconacetobacter entanii* KS542	42.0/(node 7)	63/35	*Vibrio* phage martha 12B12 (NC_021070)	*-*
			*Escherichia* phage D108 (NC_013594)	-
	24.0/(node 9)	30/21	*Enterobacter*ia phage SfI (NC_027339)	*Komagataeibacter* phage phiKM1 (LC644974.1): 95/95.5
			*Shigella* phage SfII (NC_021857)	-
*Gluconacetobacter entanii* KS544	39.4/(node 30)	56/31	*Vibrio* phage martha 12B12 (NC_021070)	-
			*Escherichia* phage D108 (NC_013594)	-
*Gluconacetobacter entanii* KS545	42.1/(node 7)	63/35	*Vibrio* phage martha 12B12 (NC_021070)	-
			*Escherichia* phage D10 (NC_013594)	-

**Table 8:** KOs that exist in all *Gluconacetobacter entanii* strains (*n* = 9) and do not exist in any *Novacetimonas* species (*n* = 15).

KO	Definition
K03273	gmhB; D-glycero-D-manno-heptose 1,7-bisphosphate phosphatase [EC:3.1.3.82 3.1.3.83]
K09732	K09732; uncharacterized protein
K22305	psp; phosphoserine phosphatase [EC:3.1.3.3]

**Table 9:** KOs that exist in 75% of the *Gluconacetobacter entanii* strains (*n* = 9) and do not exist in more than 25% of *Novacetimonas* strains (*n* = 15).

KO	Definition
K03273	GmhB; D-glycero-D-manno-heptose 1,7-bisphosphate phosphatase [EC:3.1.3.82 3.1.3.83]
K07733	AlpA; prophage regulatory protein
K09732	K09732; uncharacterized protein
K14414	RtcR; transcriptional regulatory protein RtcR
K22305	Psp; phosphoserine phosphatase [EC:3.1.3.3]
K23123	PxpB; 5-oxoprolinase (ATP-hydrolysing) subunit B [EC:3.5.2.9]

## Data Availability

Data is contained within the article.

## Supplementary Materials

The following supporting information can be downloaded at: https://www.mdpi.com/article/10.3390/foods12010214/s1, Table S1: The overall genome similarities (%) between *G. entanii* strains calculated by orthologous average nucleotide identity algorithm (OrthoANI); Table S2: In silico DNA-DNA hybridization analysis between genomes of *G. entanii* strains. The similarities were calculated by the Genome-to-Genome Distance Calculator 2.1; Table S3: Distribution of different Rep proteins from *Novacetimonas*, *Komagataeibacter*, and *Acetobacter* plasmids in genomes of *Gluconacetobacter entanii* strains; Table S4: KOs overrepresented in the *G. entanii* species group when compared to all other *Novacetimonas* species. Values in the table correspond to median gene/KO copy number values; Table S5: KOs underrepresented in the *G. entanii* species when compared to all other *Novacetimonas* species. Values in the table correspond to median gene/KO copy number values. Figure S1: Aligned draft bacterial genomes of *G. entanii* AV429, *G. entanii* AV429-2020 and *G. entanii* AV429-2022 by Mauve. Each of the draft genome sequences was at the beginning aligned to a reference chromosome sequence *Novacetimonas hansenii* C110 (acc. no. CP062147). The matches are partitioned into a minimum set of collinear blocks. Each sequence of identically colored blocks represents a collinear set of matching regions. One connecting line is drawn per collinear block.
