# Supplementary material for: Comparative Genomics and Phenotypic Characterization of Gluconacetobacter entanii, a Highly Acetic Acid-Tolerant Bacterium from Vinegars
Source: Foods. 2023 Jan 3;12(1):214. doi: 10.3390/foods12010214 (PMC9818992; doi:10.3390/foods12010214)
Supplement: Supplementary file 1 [file foods-12-00214-s001.zip › foods-2080747-supplementary.pdf]

Table S1. The overall genome similarities (%) between *G. entanii* strains calculated by orthologous average nucleotide identity algorithm (OrthoANI).

	***G. entanii* AV429**	***G. entanii* FXV2**	***G. entanii* KS542**	***G. entanii* KS544**	***G. entanii* KS545**	***G. entanii* SI2084**
***G. entanii* AV429**	/	98.60	98.34	98.31	98.32	98.33
***G. entanii* FXV2**	98.60	/	98.50	98.51	98.52	98.48
***G. entanii* KS542**	98.34	98.50	/	100.00	99.99	99.99
***G. entanii* KS544**	98.31	98.51	100.00	/	99.99	99.98
***G. entanii* KS545**	98.32	98.52	99.99	99.99	/	99.99
***G. entanii* SI2084**	98.33	98.48	99.99	99.98	99.99	/

Table S2. *In silico* DNA-DNA hybridization analysis between genomes of *G. entanii* strains. The similarities were calculated by the Genome-to-Genome Distance Calculator 2.1.

	G. entanii AV429 (%)	*G. entanii* SI2084 (%)	*G. entanii* KS545 (%)	*G. entanii* KS544 (%)	*G. entanii* KS542 (%)	*G. entanii* FXV2 (%)
G. entanii AV429	100.0					
G. entanii SI2084	77.5	100.0				
G. entanii KS545	77.8	99.7	100.0			
G. entanii KS544	77.6	99.9	99.9	100.0		
G. entanii KS542	77.7	99.9	99.9	100.0	100.0	
G. entanii FXV2	88.4	78.9	78.3	79.2	79.2	100.0

Table S3. Distribution of different Rep proteins from *Novacetimonas*, *Komagataeibacter*, and *Acetobacter* plasmids in genomes of *Gluconacetobacter entanii* strains.

Plasmid (Acc. No.)	Rep protein (Acc. No.)	*G. entanii* LTH4560^T	*G. entanii* AV429	*G. entanii* SI2084	*G. entanii* FXV2
CP062148	QOF96883.1	+	+	+	-
AJ223503	CAA11421.1	+	+	-	-
AP012160	BAK85968.1	+	+	+	+
AP012161	BAK85224.1	+	+	+	-
AP012162	BAK85302.1	+	+	+	+
CP019877	AQU89268.1	+	+	+	-
CP019878	AQU89325.1	+	+	+	+
CP019879	AQU89344.1	+	+	+	+
CP023037	AXY23809.1	-	-	+	-
CP023038 (type 1)	AXY24020.1	-	-	+	-
CP023038 (type 2)	AXY24036.1	+	-	-	-
CP023039	AXY24109.1	+	-	-	-
CP023040	AXY24276.1	+	-	-	-
CM018731	KAB8122177.1	+	-	-	-
LT575494	SAY50089.1	-	-	+	-
CP004361	AHI27136.1	+	-	-	-
CP004362	AHI27138.1	+	+	-	-
CP004363	AHI27167.1	+	-	-	-
CP004364	AHI27256.1	+	+	+	+
CP039845	QHM90155.1	-	-	-	-
LN606601	CEF43042.1	+	+	+	+
CP043507 (type 1)	QEO18808.1	+	-	-	-
CP011123	ANA15375.1	+	+	+	+
LN609303 (type 1)	CEF57342.1	+	-	-	-
LN609304	CEF57494.1	+	+	+	+
AP018516 (type 1)	BBC81753.1	+	-	-	-
CP014688	AQT06463.1	+	-	-	-
CP030873	AXC27928.1	+	+	+	+
CP023190	AXN01876.1	+	-	-	-
AP023412	BCK77757.1	+	+	+	+

CP021925	ASC07527.1	+	+	+	+
CP015169 (type 1)	AOW50630.1	+	+	+	+
AP011122 (type 1)	BAI00816.1	+	-	-	-
AP011124	BAI01161.1	+	+	+	+
AP014882 (type 1)	BAU39745.1	+	-	-	-
AP014885	BAU39956.1	+	-	-	-
HF677572	CCT61022.1	+	+	+	-
HF677573	CCT61029.1	+	+	+	+
HF677576	CCT61057.1	+	-	-	-
Sum of Rep proteins		35	20	21	14

Table S4. KOs overrepresented in the *G. entanii* species group when compared to all other *Novacetimonas* species. Values in the table correspond to median gene/KO copy number values.

ID	*G. entanii* (n=9)	*Novacetimonas* spp. (n=15)	difference	Definition
**K03496**	6	2	4	parA, soj; chromosome partitioning protein
**K00135**	8	5	3	gabD; succinate-semialdehyde dehydrogenase / glutarate-semialdehyde dehydrogenase [EC:1.2.1.16 1.2.1.79 1.2.1.20]
**K03497**	6	3	3	parB, spo0J; ParB family transcriptional regulator, chromosome partitioning protein
**K03530**	6	3	3	hupB; DNA-binding protein HU-beta
**K01153**	3	1	2	hsdR; type I restriction enzyme, R subunit [EC:3.1.21.3]
**K04763**	4	2	2	xerD; integrase/recombinase XerD
**K18201**	2	0	2	AGPHD1; hydroxylysine kinase [EC:2.7.1.81]
**K22616**	2	0	2	olsC; ornithine lipid ester-linked acyl 2-hydroxylase [EC:1.14.11.58]
**K00064**	1	0	1	E1.1.1.122; D-threo-aldose 1-dehydrogenase [EC:1.1.1.122]
**K00077**	2	1	1	panE, apbA; 2-dehydropantoate 2-reductase [EC:1.1.1.169]
**K00111**	2	1	1	glpA, glpD; glycerol-3-phosphate dehydrogenase [EC:1.1.5.3]
**K00146**	1	0	1	feaB, tynC; phenylacetaldehyde dehydrogenase [EC:1.2.1.39]
**K00151**	1	0	1	hpaE, hpcC; 5-carboxymethyl-2-hydroxymuconic-semialdehyde dehydrogenase [EC:1.2.1.60]
**K00154**	1	0	1	E1.2.1.68; coniferyl-aldehyde dehydrogenase [EC:1.2.1.68]
**K00156**	1	0	1	poxB; pyruvate dehydrogenase (quinone) [EC:1.2.5.1]

**K00330**	2	1	1	nuoA; NADH-quinone oxidoreductase subunit A [EC:7.1.1.2]
**K00331**	2	1	1	nuoB; NADH-quinone oxidoreductase subunit B [EC:7.1.1.2]
**K00334**	2	1	1	nuoE; NADH-quinone oxidoreductase subunit E [EC:7.1.1.2]
**K00335**	2	1	1	nuoF; NADH-quinone oxidoreductase subunit F [EC:7.1.1.2]
**K00336**	2	1	1	nuoG; NADH-quinone oxidoreductase subunit G [EC:7.1.1.2]
**K00337**	2	1	1	nuoH; NADH-quinone oxidoreductase subunit H [EC:7.1.1.2]
**K00339**	2	1	1	nuoJ; NADH-quinone oxidoreductase subunit J [EC:7.1.1.2]
**K00340**	2	1	1	nuoK; NADH-quinone oxidoreductase subunit K [EC:7.1.1.2]
**K00341**	2	1	1	nuoL; NADH-quinone oxidoreductase subunit L [EC:7.1.1.2]
**K00342**	2	1	1	nuoM; NADH-quinone oxidoreductase subunit M [EC:7.1.1.2]
**K00343**	2	1	1	nuoN; NADH-quinone oxidoreductase subunit N [EC:7.1.1.2]
**K00344**	3	2	1	qor, CRYZ; NADPH:quinone reductase [EC:1.6.5.5]
**K00433**	1	0	1	cpo; non-heme chloroperoxidase [EC:1.11.1.10]
**K00446**	1	0	1	dmpB, xylE; catechol 2,3-dioxygenase [EC:1.13.11.2]
**K00483**	1	0	1	hpaB; 4-hydroxyphenylacetate 3-monooxygenase [EC:1.14.14.9]
**K00549**	2	1	1	metE; 5-methyltetrahydropteroyltriglutamate--homocysteine methyltransferase [EC:2.1.1.14]
**K00558**	1	0	1	DNMT1, dcm; DNA (cytosine-5)-methyltransferase 1 [EC:2.1.1.37]
**K00782**	2	1	1	lldG; L-lactate dehydrogenase complex protein LldG
**K00839**	2	1	1	pucG; (S)-ureidoglycine---glyoxylate transaminase [EC:2.6.1.112]
**K00864**	2	1	1	glpK, GK; glycerol kinase [EC:2.7.1.30]
**K01092**	3	2	1	E3.1.3.25, IMPA, suhB; myo-inositol-1(or 4)-monophosphatase [EC:3.1.3.25]
**K01322**	1	0	1	PREP; prolyl oligopeptidase [EC:3.4.21.26]
**K01568**	2	1	1	PDC, pdc; pyruvate decarboxylase [EC:4.1.1.1]
**K01689**	2	1	1	ENO, eno; enolase [EC:4.2.1.11]
**K01703**	2	1	1	leuC, IPMI-L; 3-isopropylmalate/(R)-2-methylmalate dehydratase large subunit [EC:4.2.1.33 4.2.1.35]
**K01826**	1	0	1	hpaF, hpcD; 5-carboxymethyl-2-hydroxymuconate isomerase [EC:5.3.3.10]
**K01961**	3	2	1	accC; acetyl-CoA carboxylase, biotin carboxylase subunit [EC:6.4.1.2 6.3.4.14]
**K01971**	1	0	1	ligD; bifunctional non-homologous end joining protein LigD [EC:6.5.1.1]
**K02013**	4	3	1	ABC.FEV.A; iron complex transport system ATP-binding protein [EC:7.2.2.-]
**K02015**	4	3	1	ABC.FEV.P; iron complex transport system permease protein
**K02016**	4	3	1	ABC.FEV.S; iron complex transport system substrate-binding protein
**K02160**	3	2	1	accB, bccP; acetyl-CoA carboxylase biotin carboxyl carrier protein

**K02232**	2	1	1	cobQ, cbiP; adenosylcobyric acid synthase [EC:6.3.5.10]
**K02274**	2	1	1	coxA, ctaD; cytochrome c oxidase subunit I [EC:7.1.1.9]
**K02275**	1	0	1	coxB, ctaC; cytochrome c oxidase subunit II [EC:7.1.1.9]
**K02276**	1	0	1	coxC, ctaE; cytochrome c oxidase subunit III [EC:7.1.1.9]
**K02440**	2	1	1	GLPF; glycerol uptake facilitator protein
**K02509**	1	0	1	hpaH; 2-oxo-hept-3-ene-1,7-dioate hydratase [EC:4.2.1.-]
**K02510**	1	0	1	hpaI, hpcH; 4-hydroxy-2-oxoheptanedioate aldolase [EC:4.1.2.52]
**K03111**	2	1	1	ssb; single-strand DNA-binding protein
**K03299**	5	4	1	TC.GNTP; gluconate:H+ symporter, GntP family
**K03442**	1	0	1	mscS; small conductance mechanosensitive channel
**K03559**	3	2	1	exbD; biopolymer transport protein ExbD
**K03561**	3	2	1	exbB; biopolymer transport protein ExbB
**K03762**	1	0	1	proP; MFS transporter, MHS family, proline/betaine transporter
**K03777**	2	1	1	dld; D-lactate dehydrogenase (quinone) [EC:1.1.5.12]
**K03832**	3	2	1	tonB; periplasmic protein TonB
**K04047**	1	0	1	dps; starvation-inducible DNA-binding protein
**K05343**	1	0	1	treS; maltose alpha-D-glucosyltransferase / alpha-amylase [EC:5.4.99.16 3.2.1.1]
**K05349**	3	2	1	bglX; beta-glucosidase [EC:3.2.1.21]
**K05595**	2	1	1	marC; multiple antibiotic resistance protein
**K05896**	3	2	1	scpA; segregation and condensation protein A
**K06199**	1	0	1	crcB, FEX; fluoride exporter
**K07126**	2	1	1	K07126; uncharacterized protein
**K07152**	3	2	1	SCO1; protein SCO1
**K07160**	2	1	1	pxpA; 5-oxoprolinase (ATP-hydrolysing) subunit A [EC:3.5.2.9]
**K07221**	2	1	1	oprO_P; phosphate-selective porin OprO and OprP
**K07452**	1	0	1	mcrB; 5-methylcytosine-specific restriction enzyme B [EC:3.1.21.-]
**K07484**	1	0	1	K07484; transposase
**K07488**	1	0	1	K07488; transposase
**K07507**	1	0	1	mgtC; putative Mg2+ transporter-C (MgtC) family protein
**K07789**	3	2	1	mdtC; multidrug efflux pump
**K08309**	3	2	1	slt; soluble lytic murein transglycosylase [EC:4.2.2.-]
**K08995**	2	1	1	K08995; putative membrane protein

**K09024**	3	2	1	rutF; flavin reductase [EC:1.5.1.-]
**K10680**	2	1	1	nemA; N-ethylmaleimide reductase [EC:1.-.-.-]
**K10947**	1	0	1	padR; PadR family transcriptional regulator, regulatory protein PadR
**K12203**	1	0	1	dotB, traJ; defect in organelle trafficking protein DotB [EC:7.2.4.8]
**K12204**	1	0	1	dotC, traI; defect in organelle trafficking protein DotC
**K12217**	1	0	1	icmO, trbC, dotL; intracellular multiplication protein IcmO [EC:7.2.4.8]
**K12218**	1	0	1	icmP, trbA; intracellular multiplication protein IcmP
**K13993**	1	0	1	HSP20; HSP20 family protein
**K16092**	2	1	1	btuB; vitamin B12 transporter
**K16147**	1	0	1	glgE; starch synthase (maltosyl-transferring) [EC:2.4.99.16]
**K18843**	1	0	1	hicB; antitoxin HicB
**K18928**	2	1	1	lldE; L-lactate dehydrogenase complex protein LldE
**K18929**	2	1	1	lldF; L-lactate dehydrogenase complex protein LldF
**K18954**	1	0	1	pobR; AraC family transcriptional regulator, transcriptional activator of pobA
**K19147**	1	0	1	mcrC; 5-methylcytosine-specific restriction enzyme subunit McrC
**K19837**	1	0	1	atzE; 1-carboxybiuret hydrolase [EC:3.5.1.131]
**K21495**	1	0	1	fitA; antitoxin FitA
**K22473**	2	1	1	adhA; alcohol dehydrogenase (quinone), dehydrogenase subunit [EC:1.1.5.5]
**K22895**	4	3	1	cruH; renierapurpurin 18,18'-hydroxylase
**K23124**	2	1	1	pxpC; 5-oxoprolinase (ATP-hydrolysing) subunit C [EC:3.5.2.9]

Table S5. KOs underrepresented in the *G. entanii* species when compared to all other *Novacetimonas* species. Values in the table correspond to median gene/KO copy number values.

**ID**	***G. entanii*** **(n=9)**	***Novacetimonas*** **spp. (n=15)**	**Difference**	**Definition**
**K02014**	24	29	-5	TC.FEV.OM; iron complex outermembrane recepter protein
**K00059**	3	5	-2	fabG, OAR1; 3-oxoacyl-[acyl-carrier protein] reductase [EC:1.1.1.100]
**K02433**	2	4	-2	gatA, QRSL1; aspartyl-tRNA(Asn)/glutamyl-tRNA(Gln) amidotransferase subunit A [EC:6.3.5.6 6.3.5.7]
**K13771**	3	5	-2	nsrR; Rrf2 family transcriptional regulator, nitric oxide-sensitive transcriptional repressor
**K00027**	1	2	-1	ME2, sfcA, maeA; malate dehydrogenase (oxaloacetate-decarboxylating) [EC:1.1.1.38]

**K00117**	2	3	-1	gcd; quinoprotein glucose dehydrogenase [EC:1.1.5.2]
**K00459**	1	2	-1	ncd2, npd; nitronate monooxygenase [EC:1.13.12.16]
**K00694**	2	3	-1	bcsA; cellulose synthase (UDP-forming) [EC:2.4.1.12]
**K00799**	3	4	-1	GST, gst; glutathione S-transferase [EC:2.5.1.18]
**K00820**	1	2	-1	glmS, GFPT; glutamine---fructose-6-phosphate transaminase (isomerizing) [EC:2.6.1.16]
**K01114**	1	2	-1	plc; phospholipase C [EC:3.1.4.3]
**K01214**	1	2	-1	ISA, treX; isoamylase [EC:3.2.1.68]
**K01465**	2	3	-1	URA4, pyrC; dihydroorotase [EC:3.5.2.3]
**K01652**	2	3	-1	E2.2.1.6L, ilvB, ilvG, ilvI; acetolactate synthase I/II/III large subunit [EC:2.2.1.6]
**K01714**	1	2	-1	dapA; 4-hydroxy-tetrahydrodipicolinate synthase [EC:4.3.3.7]
**K01733**	1	2	-1	thrC; threonine synthase [EC:4.2.3.1]
**K02483**	5	6	-1	K02483; two-component system, OmpR family, response regulator
**K03307**	2	3	-1	TC.SSS; solute:Na+ symporter, SSS family
**K03457**	1	2	-1	TC.NCS1; nucleobase:cation symporter-1, NCS1 family
**K03466**	1	2	-1	ftsK, spoIIIE; DNA segregation ATPase FtsK/SpoIIIE, S-DNA-T family
**K03566**	1	2	-1	gcvA; LysR family transcriptional regulator, glycine cleavage system transcriptional activator
**K03585**	1	2	-1	acrA, mexA, adeI, smeD, mtrC, cmeA; membrane fusion protein, multidrug efflux system
**K04091**	1	2	-1	ssuD, msuD; alkanesulfonate monooxygenase [EC:1.14.14.5 1.14.14.34]
**K06221**	1	2	-1	dkgA; 2,5-diketo-D-gluconate reductase A [EC:1.1.1.346]
**K07552**	2	3	-1	bcr, tcaB; MFS transporter, DHA1 family, multidrug resistance protein
**K12373**	2	3	-1	HEXA_B; hexosaminidase [EC:3.2.1.52]
**K15553**	2	3	-1	ssuA; sulfonate transport system substrate-binding protein
**K15554**	2	3	-1	ssuC; sulfonate transport system permease protein
**K15555**	2	3	-1	ssuB; sulfonate transport system ATP-binding protein [EC:7.6.2.14]
**K18903**	1	2	-1	oprC, opcM; outer membrane protein, multidrug efflux system
**K22474**	2	3	-1	adhB; alcohol dehydrogenase (quinone), cytochrome c subunit [EC:1.1.5.5]
**K22736**	1	2	-1	VIT; vacuolar iron transporter family protein

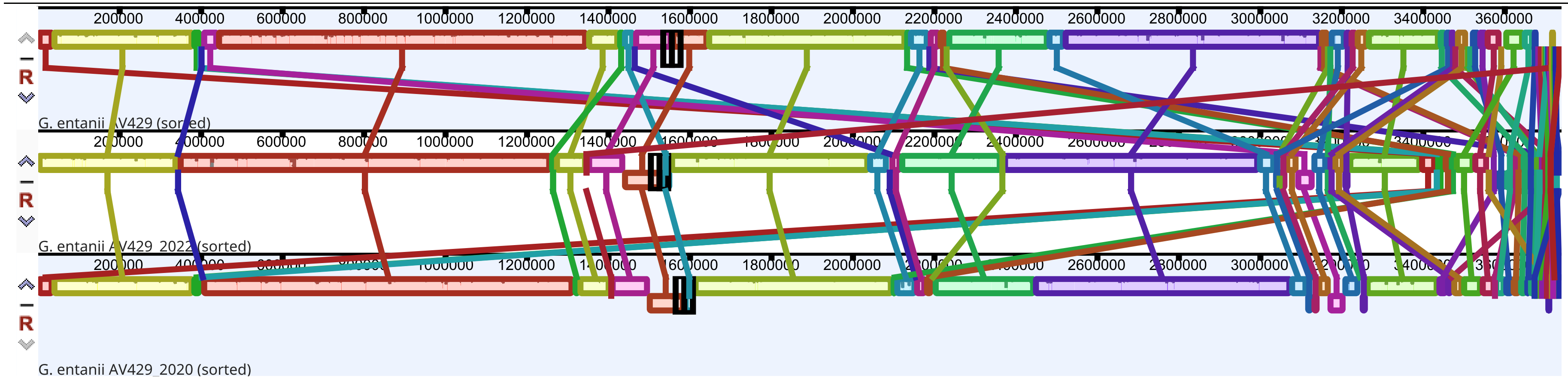

Figure S1. Aligned draft bacterial genomes of *G. entanii* AV429, *G. entanii* AV429-2020 and *G. entanii* AV429-2022 by Mauve. Each of the draft genome sequences was at the beginning aligned to a reference chromosome sequence *Novacetimonas hansenii* C110 (acc. no. CP062147). The matches are partitioned into a minimum set of collinear blocks. Each sequence of identically colored blocks represents a collinear set of matching regions. One connecting line is drawn per collinear block.
